# Editorial: Paediatric drug development

**DOI:** 10.3389/fmed.2026.1991290

**Published:** 2026-10-05

**Authors:** Mette Due Theilade Thomsen, Gesine Bejeuhr, Angeliki Siapkara, Cécile Ollivier, Edit Muhari-Stark, Mark Turner

**Affiliations:** 1 PIP Adviser ApS, Kongens Lyngby, Denmark; 2 Bayer AG, Berlin, Germany; 3 AstraZeneca, Cambridge, United Kingdom; 4 Critical Path Institute, Amsterdam, Netherlands; 5 PedMed Consulting, Chapel Hill, NC, United States; 6 University of Liverpool, Liverpool, United Kingdom

**Keywords:** childhood systemic lupus erythematosus, ICH S11, innovative health initiative, juvenile animal study, paediatric clinical trials, paediatric investigation plans, paediatric regulation, weight-of-evidence approach

The European Paediatric Regulation includes a requirement for medicine developers for a paediatric investigation plan (PIP) agreed by the European Medicines Agency (EMA) Paediatric Committee (PDCO). Learnings from the past 20 years with the Regulation have now been translated into the revised pharmaceutical legislation. This Research Topic presents 7 papers from 2025 exploring the development in regulatory requirements for PIPs and regulatory advances for paediatric drug development in Europe and worldwide. With authors from authorities, industry and academia, a wide range of topics are covered, such as the ICH S11 guideline impact on non-clinical studies to support paediatric development, EMA's views on PIPs for childhood systemic lupus erythematosus (cSLE), challenges with paediatric randomised controlled trials (RCTs), regulatory and ethics committee requirements for paediatric trials across jurisdictions, projects with paediatric impact in the Innovative Health Initiative (IHI), and the conect4children (c4c) network and its Trial Support Services.

***Views*** ***from authorities**:* The 2020 ICH S11 guideline on nonclinical safety studies to support paediatric drug development guides on juvenile animal studies (JAS) and proposes a weight-of-evidence (WoE) approach to decide on the need for such studies. In Tavares et al., experts from EMA, PDCO, the Dutch and Belgian Medicines Agencies study the guideline impact on nonclinical development for paediatric medicines. They analyse JAS proposals and their regulatory assessment in PIPs agreed between 2020 and 2023. While in 75% of cases, the PDCO agreed on the WoE assessment and non-clinical strategy proposed by the company, in some cases the company was asked to change the design of a JAS, or to include or exclude a JAS from the PIP. The authors conclude that ICH S11 facilitates science-driven discussions on the need for and design of JAS; the WoE approach ensures that relevant safety information is generated to support paediatric drug development while balancing the principles of non-clinical replacement, reduction and refinement (the 3Rs).

De Lisa et al. provide insight on EMAs experience with PIPs for cSLE and adds important input from the Paediatric Rheumatology International Trials Organisation (PRINTO) on key points in cSLE medicines development, as PRINTO has developed and validated a core set of cSLE disease severity measures. cSLE and adult SLE (aSLE) have similar pathophysiology, but the disease course of cSLE is typically more severe with frequent kidney and neuropsychiatric involvement and fast organ damage. The 22 different cSLE PIPs approved to date all include at least one double blind RCT. However, extrapolation from adult studies may be possible in cSLE and could support regulatory approval of a cSLE indication. To facilitate extrapolation, the same outcome measures used in adults should be applied in children. An acceptable alternative to a RCT could be an extrapolation plan comprising an open label pharmacokinetics trial with pharmacodynamic endpoints as a platform registry, modelling and simulation, and extrapolation of efficacy from adult trial results, and long-term safety extension studies with more patients.

***Paediatric trials:*** Moving further into the topic of paediatric clinical trials (CTs), Nebeska et al. analyse paediatric RCTs registered in Clinicaltrials.gov between 2011 and 2013 and the following publication status of these trials as found in PubMed and Google Scholar. The study highlights significant challenges in paediatric RCTs, including high incompletion rates and delays in result reporting. The authors conclude that strengthened regulatory oversight and enhanced compliance are essential to improve paediatric research outcomes and ensure timely dissemination of RCT results.

Paediatric CTs are further discussed in two interesting publications examining differences in regulatory and ethics committee (EC) requirements across jurisdictions. Enrolling paediatric participants in trials across multiple sites in different jurisdictions is particularly challenging when each jurisdiction has separate Competent Authority requirements. Stewart et al. present current legislative and regulatory requirements for CT applications including any specific for paediatrics for each of six jurisdictions: EU, United Kingdom, United States, Canada, Japan, and Australia. The work was done by a European Network of Paediatric Research at the European Medicines Agency (EnprEMA) working group aiming to facilitate regulatory harmonisation and international alignment of paediatric CTs. One further work from Stewart et al. highlights the differences across the same six jurisdictions in EC review for CTs in paediatric medicine development. While there is a growing trend for centralised ethics review, it is not universally permitted in all jurisdictions, due to institutional, state/provincial, or national policies.

***IHI paediatric research****:* Seigneuret (2025) provides useful insights from IHI and its predecessor, the Innovative Medicines Initiative (IMI), on European public-private partnership projects aiming to advance paediatric research. Projects improving paediatric clinical trials are **c4c** (now **c4c Stichting**), a pan-European paediatric clinical trial network supporting clinical trials with regulatory purpose; **EU-PEARL**, a framework and tools for patient-centric collaborative platform trials in Europe, leveraged by **RealiseD** developing playbooks for use in rare paediatric disease trials; **INNODIA** and **INNODIA HARVEST,** a clinical network on type 1 diabetes to perform complex trials; and **AIMS-2-TRIALS** studying autism development and identifying clinical trial biomarkers for clinical validation and regulatory qualification.

With the revised EU paediatric legislation, almost all new cancer medicines for adults must be explored for potential paediatric indications; the **ITCC-P4** project develops a preclinical testing platform to determine “target actionability” and xenograft models of paediatric solid tumours. **Screen4Care** aims to accelerate rare disease diagnosis via a comprehensive genetic newborn screening framework suited for healthcare system implementation. **EDENT1FI** sets up a pan-European open platform to screen 200,000 children to identify early-stage Type 1 diabetes mellitus. Immunisation projects include **RESCEU** showing that vaccinating pregnant women or healthy babies could reduce the healthcare burden caused by respiratory syncytial virus, and **PERISCOPE** generating tools to accelerate development of next-generation pertussis vaccines.

IHI/IMI projects advance European paediatric research through a range of achievements; the unique platform brings together industry, patients, academia, healthcare providers and regulators to address hurdles with cross-cutting solutions for the benefit of children.

Attar et al. discuss the c4c network Trial Support Services developed in alignment with EnprEMA. The model can potentially advance paediatric drug development by supporting faster and more efficient clinical trials while maintaining high standards of quality and compliance.

